# Comparing Antimicrobial Use Across Three Healthcare Facilities in Ekiti State, Nigeria: A Global Point Prevalence Survey

**DOI:** 10.1017/ash.2025.276

**Published:** 2025-09-24

**Authors:** Victor Amaechi Onwu, Oluwatoyosi Eniola Tanimowo, Kester Chika Agboli-Peters

**Affiliations:** 1 Ministry of Health and Human Services, Ekiti State Government, Ado Ekiti, Ekiti State, Nigeria; 2 Nigeria Centre for Disease Control, Abuja; 3 Ekiti State Primary Health Care Development Agency

## Abstract

**Background:** Antimicrobial resistance (AMR) is one of the most pressing health challenges of our time, fueled by the widespread misuse and overuse of antibiotics. Tackling this issue requires accurate, real-world data on how antimicrobials are prescribed and used. Point Prevalence Surveys (PPS) have become invaluable in this effort, offering a clear picture of prescribing practices and guiding the development of effective stewardship programs. This study focuses on antimicrobial use in three healthcare facilities in Ekiti State, Nigeria, leveraging the Global-PPS methodology to uncover patterns, pinpoint gaps, and identify opportunities to improve prescribing practices and support the fight against AMR. **Method:** This study took a hands-on approach to understanding antimicrobial prescribing practices in Ekiti State by using the well-established Global-PPS protocol. Three healthcare facilities - one each from the tertiary, secondary, and primary levels-were carefully selected to provide a broad view of prescribing behaviors. Data were gathered using standardized tools to capture key details such as patient demographics, reasons for antimicrobial use, prescribing patterns, and adherence to clinical guidelines. Descriptive statistics were used to summarize the trends, while comparisons across the facilities highlighted important differences. To ensure the findings were practical and relevant, we worked closely with the relevant MDAs, fostering a collaborative effort that added depth and context to the study. **Results:** Preliminary findings revealed significant variations in antimicrobial prescribing patterns, with the tertiary facility showing 75% adherence to stewardship protocols, compared to 45% and 30% in secondary and primary centers, respectively. Factors contributing to inappropriate prescriptions included limited diagnostic access (tertiary - 85%, secondary - 50%, primary - 25%), inadequate guideline dissemination (tertiary - 90%, secondary - 40%, primary - 20%), and insufficient prescriber training. Empirical therapy without justification was common, accounting for 60% of cases in secondary and 75% in primary centers. These gaps underscore the need for targeted interventions to improve prescribing practices. **Conclusion:** This study highlights the pressing need for customized antimicrobial stewardship programs in Ekiti State, Nigeria. By shedding light on prescribing habits and identifying critical gaps, these findings pave the way for meaningful, locally relevant interventions that encourage responsible use of antibiotics. Strengthening healthcare capacity, expanding access to diagnostic tools, and fostering adherence to treatment guidelines are essential next steps. These efforts not only hold the promise of improving patient care in Ekiti State but also contribute to the broader fight against antimicrobial resistance.

